# Patients undergoing multiligament knee reconstruction injured during pivoting sports demonstrate similar clinical, functional and return to sport outcomes by 2 years compared with those undergoing anterior cruciate ligament reconstruction

**DOI:** 10.1002/ksa.12409

**Published:** 2024-08-05

**Authors:** Jay R. Ebert, Peter K. Edwards, Alistair I. W. Mayne, Peter S. E. Davies, Robert Evans, Randeep S. Aujla, Shahbaz S. Malik, Stephen Dalgleish, Satyen Gohil, Peter D'Alessandro

**Affiliations:** ^1^ School of Human Sciences (Exercise and Sport Science) University of Western Australia Perth Western Australia Australia; ^2^ HFRC Rehabilitation Clinic Perth Western Australia Australia; ^3^ Orthopaedic Research Foundation of Western Australia Perth Western Australia Australia; ^4^ School of Allied Health Curtin University Perth Western Australia Australia; ^5^ Fiona Stanley and Fremantle Hospitals Group, South Metropolitan Health Service Perth Western Australia Australia; ^6^ Joondalup Health Campus Perth Western Australia Australia; ^7^ Leicester Knee Unit University Hospitals of Leicester NHS Trust Leicester UK; ^8^ Worcestershire Acute Hospitals NHS Trust Worcester UK; ^9^ Ninewells Hospital and Medical School Dundee UK; ^10^ Orthopaedics WA, Wexford Medical Centre Perth Western Australia Australia; ^11^ Coastal Orthopaedics Perth Western Australia Australia; ^12^ School of Surgery University of Western Australia Perth Western Australia Australia

**Keywords:** anterior cruciate ligament reconstruction, clinical outcomes, knee function, multiligament knee reconstruction, return to sport

## Abstract

**Purpose:**

This study investigates the clinical and activity‐based outcomes after anterior cruciate ligament reconstruction (ACLR) versus multiligamentous knee reconstruction (MLKR) following a pivoting sports injury.

**Methods:**

Fifty MLKR patients were included, of which 20 (40%) were injured during pivoting sports. A further 50 patients undergoing ACLR following an injury during pivoting sports were consecutively recruited for comparison. Patients were assessed before the surgery and at 6‐, 12‐ and 24 months with patient‐reported outcome measures (PROMs) including the International Knee Documentation Committee (IKDC) form, Tegner activity scale (TAS) and anterior cruciate ligament return to sport after injury (ACL‐RSI) score. Knee movement, the single (SHD) and triple (THD) hop tests for distance, and peak isokinetic knee extensor and flexor strength were assessed, with Limb Symmetry Indices (LSIs) calculated. Outcomes were compared across groups: (1) ACLR (*n* = 50), (2) MLKR (*n* = 50) and (3) MLKR due to pivoting sport injury (*n* = 20).

**Results:**

IKDC, TAS and ACL‐RSI scores remained lower (*p* < 0.05) in the full MLKR versus ACLR cohort at all timepoints. Comparing the ACLR and MLKR cohort that had injuries specifically during pivoting sports, the IKDC (*p* < 0.001) and TAS (*p* = 0.009) were higher in the ACLR group at 6 months, and the ACL‐RSI was higher at 6 (*p* < 0.001) and 12 (*p* = 0.007) months, there were no further differences. Hop and knee extensor strength LSIs were lower (*p* < 0.05) in the full MLKR (versus ACLR) cohort at all timepoints (apart from the 24‐month SHD LSI). However, the ACLR group only demonstrated greater LSIs than the pivoting sport MLKR for the SHD at 6 months (*p* < 0.001), and knee extensor strength at 6 (*p* < 0.001) and 12 (*p* < 0.001) months.

**Conclusions:**

While the recovery of patients undergoing MLKR due to a pivoting sports injury is delayed compared with their ACLR counterparts, the clinical outcome and activity profile are similar by 24 months.

**Level of Evidence:**

Level IV.

**Abbreviations**
ACL: anterior cruciate ligament
ACLR: anterior cruciate ligament reconstruction
ACLR: anterior cruciate ligament reconstruction
ACL‐RSI: anterior cruciate ligament return to sport after injury
ANOVA: analysis of variance
GRC: global rating of change
IKDC: International Knee Documentation Committee
MCL: medial collateral ligament
MLKI: multiligamentous knee injury
MLKR: multiligament knee reconstruction
PCL: posterior cruciate ligament
PLC: posterolateral corner
PROMs: patient‐reported outcome measures
RTS: return to sport
SHD: single horizontal hop for distance
TAS: Tegner activity scale

## INTRODUCTION

The incidence of multiligamentous knee injury (MLKI) is estimated at 0.072 events per 100 person‐years [3]. In comparison, anterior cruciate ligament (ACL) injuries account for 50% of all knee injuries, with >120,000 annually in the United States alone [15]. It is estimated that ACL reconstruction (ACLR) is 60 times more common than multiligamentous reconstruction (MLKR) [39]. Nonetheless, improved clinical outcomes, as well as return to work and sports activity, have been reported in patients following MLKR [9, 13, 16, 27, 30, 32, 40].

Although the primary aim of MLKR is to restore the patient to an adequate level of daily function, many patients desire a more active lifestyle and a return to pivoting sports. Given the significant nature of MLKI, as well as the fact that published ACL research reports that only 65% of patients return to their preinjury level of sport after ACLR [2], it can be expected that return to sport (RTS) rates are lower after MLKR. However, there is a paucity of evidence reporting on strength, functional and activity‐related recovery, particularly compared with their ACLR counterparts. This information is important in more accurately counselling patients on expected recovery timeframes and providing realistic expectations.

The current study hypothesized that (1) significantly higher postoperative PROMs would be observed in ACLR patients versus those undergoing MLKR through various injury mechanisms, although no differences would be observed when comparing ACLR patients with MLKR patients injured specifically during pivoting sports, (2) 24‐month postoperative sports activity would be significantly higher in ACLR patients versus the larger, heterogeneous MLKR cohort, though no differences would be seen when comparing ACLR patients to MLKR patients injured during pivoting sports and (3) significantly higher postoperative limb symmetry indices (LSIS) for lower limb strength and hop measures would be observed between ACLR patients and the larger, heterogeneous MLKR cohort, though no differences would be seen when comparing ACLR patients to MLKR patients injured during pivoting sports.

## MATERIALS AND METHODS

A total of 54 patients consecutively presenting following MLKI were prospectively recruited and underwent MLKR, of which 50 patients were included (Table 1). Four patients were omitted due to inability to attend assessments. Of those, 20 patients (40%) injured their knee during pivoting sports (Australian Rules Football [*n* = 14], soccer [*n* = 3], basketball/netball [*n* = 2], rugby [*n* = 1]), with 30 (60%) via other mechanisms (nonpivoting sports, work or activities of daily living, motor vehicles accidents) (Table 1). A total of 50 patients who experienced ACL rupture via pivoting sports (Australian Rules Football [*n* = 19], soccer [*n* = 7], basketball/netball [*n* = 9], rugby [*n* = 7], other [*n* = 8]) and underwent ACLR (hamstring autograft with, or without, concomitant meniscal repair) were prospectively recruited (Table 1). These were not matched to MLKR patients. Both cohorts had the same preoperative PROMs collected, and postoperative PROMs and physical measures were assessed at 6, 12 and 24 months. Ethics approval was provided by the relevant human research ethics committee and all patients provided consent.

**Table 1:** Patient demographics, along with injury and surgery characteristics, of patients in the full ACLR (*n* = 50) and MLKR (*n* = 50) cohorts, as well as the MLKR cohort that injured themselves participating in pivoting sports (*n* = 20).

Variable	MLKR	MLKR (pivoting)	ACLR
*n*	50	20	50
Gender, males (%)	42 (84%)	17 (85%)	42 (84%)
Age, years	30.1 (9.1), 16–50	28.5 (8.2), 16–40	28.1 (9.1), 16–47
BMI	27.2 (4.4), 16.2–38.2	25.9 (4.4), 16.2–38.2	25.6 (3.5), 18.8–38.7
Time (injury to surgery), days	7.7 (19.1), 1–128	8.3 (9.4), 2–32	64.6 (92.6), 11–364
Injury mechanism (noncontact)	7 (14%)	4 (20%)	42 (84%)
Mechanism of injury			
Sports (pivoting)	20 (40%)	20 (100%)	50 (100%)
Sports (other)	7 (14%)	0 (0%)	0 (0%)
Work/ADLs	15 (30%)	0 (0%)	0 (0%)
MVA	8 (16%)	0 (0%)	0 (0%)
Concomitant injury/surgery			
Medial meniscal repair	13 (26%)	5 (25%)	14 (28%)
Lateral meniscal repair	11 (22%)	5 (25%)	9 (18%)
OATS	1 (2%)	0 (0%)	0 (0%)
Tibial plateau fracture	1 (2%)	0 (0%)	0 (0%)
CPN injury	7 (14%)	2 (10%)	0 (0%)
Schenck classification			
KD I	37 (74%)	17 (85%)	NA
KD II	3 (6%)	1 (5%)	NA
KD III	0 (0%)	0 (0%)	NA
KD IIIM	5 (10%)	0 (0%)	NA
KD IIIL	4 (8%)	2 (10%)	NA
KD IV	1 (2%)	0 (0%)	NA
KD V	0 (0%)	0 (0%)	NA
Ligament reconstruction combinations			
ACL/MCL	8 (16%)	6 (30%)	NA
ACL/PLC	17 (34%)	8 (40%)	NA
ACL/PMC	5 (10%)	3 (15%)	NA
PCL/MCL	5 (10%)	0 (0%)	NA
PCL/PLC	2 (4%)	0 (0%)	NA
ACL/PCL	3 (6%)	1 (5%)	NA
ACL/PCL/LCL	2 (4%)	1 (5%)	NA
ACL/PCL/MCL	4 (8%)	0 (0%)	NA
ACL/PCL/PLC	2 (4%)	1 (5%)	NA
ACL/PCL/PMC	1 (2%)	0 (0%)	NA
ACL/PCL/PLC/PMC	1 (2%)	0 (0%)	NA

Abbreviations: ACL, anterior cruciate ligament; ACLR, anterior cruciate ligament reconstruction; ADLs, activities of daily living; BMI, body mass index; CPN, common peroneal nerve; KD, knee dislocation; LCL, lateral collateral ligament; MCL, medial collateral ligament; MLKR, multiligament knee reconstruction; MVA, motor vehicle accident; NA, not applicable; OATS, osteochondral autograft transplantation; PMC, posteromedial corner; PCL, posterior cruciate ligament; PLC, posterolateral corner; PMC, posteromedial corner.

### MLKR and ACLR Surgery and Rehabilitation

MLKR procedures were undertaken by one of two experienced orthopaedic surgeons, generally as a single‐stage surgical reconstruction and concomitant repair of all injured ligaments. Preferred graft options are summarized in Table 2. In cases with combined cruciate and collateral ligament injury, cruciate ligament reconstruction was undertaken before extra‐articular reconstruction, with graft tensioning in the following order: posterior cruciate ligament (PCL), anterior cruciate ligament (ACL), posterolateral corner (PLC), medial collateral ligament (MCL). For both the PLC and MCL/posterior cruciate ligament (PMC), anatomic repair of any torn structures was first undertaken, with reattachment using soft tissue anchors. For PLC reconstruction, a lateral knee approach was performed from the lateral epicondyle to the fibular neck and a modified Arciero reconstruction was performed with two femoral tunnels. The common peroneal nerve (CPN) was identified proximally and a full neurolysis was performed in all cases. When clinical CPN injury had been preoperatively identified, the zone of injury to the nerve was measured and photographed with subsequent assessment by a peripheral nerve surgeon with consideration of nerve/tendon transfer surgery in cases without spontaneous recovery. For MCL reconstruction, included patients were considered to have grade III MCL/PMC instability requiring a full reconstruction, and a modified Lind technique was used [25]. For ACLR patients, these were performed using a hamstring semitendinosus autograft harvested from the ipsilateral limb, with gracilis harvested in some cases to ensure a minimum graft diameter of at least 8 mm in all patients. If required, concomitant surgery (e.g. meniscal repair) was performed in conjunction with ACLR.

**Table 2:** Preferred graft choices for MLKR in the current cohort.

Injury type	Grafts
ACL/MCL	ACL—ipsilateral BPTB or QT autograft
	MCL—contralateral HT autograft or achilles/peroneus longus allograft or LARS
ACL/PLC	ACL—ipsilateral HT autograft
	PLC—LARS synthetic ligament
PCL/MCL	PCL—hybrid graft (contralateral HT autograft and LARS synthetic ligament)
	MCL—peroneus longus or achilles allograft or LARS
PCL/PLC	PCL—hybrid graft (ipsilateral HT autograft and LARS synthetic ligament or peroneus longus allograft)
	PLC—LARS synthetic ligament
ACL/PCL	ACL—ipsilateral BPTB or QT autograft or contralateral HT autograft
	PCL—hybrid graft (ipsilateral HT autograft and LARS synthetic ligament)
ACL/PCL/MCL	ACL—ipsilateral BPTB or QT autograft
	PCL—hybrid graft (contralateral HT autograft and LARS synthetic ligament or peroneus longus allograft)
	MCL—peroneus longus or achilles allograft or LARS
ACL/PCL/PLC	ACL—ipsilateral HT or QT autograft
	PCL—hybrid graft (ipsilateral HT autograft and LARS synthetic ligament)
	PLC—LARS synthetic ligament
ACL/PCL/PLC/PMC	ACL—peroneus longus allograft
	PCL—hybrid graft (contralateral HT autograft and LARS synthetic ligament)
	MCL—peroneus longus or achilles allograft
	PLC—LARS synthetic ligament

Abbreviations: ACL, anterior cruciate ligament; BPTB, bone‐patella tendon‐bone; HT, hamstrings tendon; LARS, ligament augmentation reconstruction system; PCL, posterior cruciate ligament; PLC, posterolateral corner; PMC, posteromedial corner; LCL, lateral collateral ligament; MCL, medial collateral ligament; QT, quadriceps tendon.

A hinge brace was employed for 6 weeks in MLKR patients, though a Jack dynamic PCL brace (Ossur) was used for 12 weeks in PCLR patients. Knee bracing was locked in extension for ambulation, though 90° of knee flexion was permitted for nonweightbearing ROM exercises. Patients were typically partial weight‐bearing with crutches for 6 weeks, then progressing to full weight‐bearing. ACLR patients were permitted full weight‐bearing as tolerated, though bracing and restricted early weight‐bearing was advocated with concomitant meniscal repair. Although meniscal repair rarely affected the progression of rehabilitation in those undergoing MLKR, in ACLR patients the weight‐bearing status was modified with a gradual reduction in crutch use and bracing over 6 weeks. Unstable meniscal tears (radial tears) were treated with up to 6 weeks of restricted weight bearing, as deemed appropriate by the treating specialist, with stable meniscal tears permitted to weight bear immediately as tolerated. After 6 weeks, the presence of concomitant meniscal repair did not affect rehabilitation.

For both MLKR and ACLR cohorts, outside of the early guidance surrounding bracing and weight‐bearing restrictions, rehabilitation was not standardized. This was under the direction of the patient's out‐patient therapist, in collaboration with the orthopaedic team, further dictated by the patient's underlying conditioning, response to exercises and readiness to proceed throughout the postoperative timeline. Furthermore, the later stage initiation of higher‐level loading, plyometric, agility, and/or sport‐specific training activities where relevant was not standardized, nor were the criteria in place to determine an individual's RTS. However, a multidisciplinary approach was adopted as part of this decision‐making process in both cohorts.

### Clinical review

PROMs were completed presurgery and at 6, 12 and 24 months. These included the International Knee Documentation Committee (IKDC) Subjective Knee Evaluation form [18], the Lysholm knee score (LKS) [26], the Cincinnati knee rating system (CKRS) [6] and the knee outcome survey (KOS) [19]. The preinjury (and postsurgery) Tegner activity scale (TAS) [35] was completed. At 6, 12 and 24 months, the anterior cruciate ligament return to sport after injury (ACL‐RSI) scale [37] was completed, as was a global rating of change (GRC) scale, scored from −5 (very much worse) to 0 (about the same) to 5 (completely recovered). Satisfaction with the surgical outcome was assessed, as was satisfaction with pain relief, and the ability to perform normal daily, recreational (walking, swimming, cycling, golf, dancing) and sports (including sports such as tennis, netball, soccer and football) activities. At 6, 12 and 24 months, active knee flexion and extension range of motion (ROM) was evaluated. The single (SHD) and triple (THD) hop tests for distance were assessed [33], as were peak isokinetic knee extensor and flexor torque using an isokinetic dynamometer (Isosport International) at an isokinetic speed of 90°/s.

### Data and statistical analysis

First, LSIs were calculated for the hop and strength measures. The mean (standard deviation [SD]) of all pre‐ and postoperative PROMs, knee ROM and physical performance LSIs were presented. Specific to the full MLKR cohort, repeated measures analysis of variance (ANOVA) was employed to investigate PROMs over time between KDI and KDII‐V groups, as well as between those that did, and did not, have concomitant CPN involvement. Specific to the ACLR cohort, repeated measures ANOVA was employed to evaluate the presence of any differences in PROMs between those that did, or did not, undergo concomitant meniscal repair. There were no differences (n.s.) in any outcome measure, hence the ACLR group was assessed collectively. ANOVA was then employed to assess differences across groups (the ACLR group, the full MLKR group, and the MLKR group that injured their knee during pivoting sports) over time in all subjective PROMs and objective measures. Where a significant group or interaction effect was found, post hoc independent *t* tests were used to determine timepoints at which the groups differed. For all three groups, the number (and percentage) of patients reporting ‘Very Satisfied’, ‘Somewhat Satisfied’, ‘Somewhat Dissatisfied’ and ‘Very Dissatisfied’ within each of the satisfaction domains at 24 months was presented. Statistical analysis was performed using SPSS software (SPSS version 27.0; SPSS Inc.), with significance determined at *p* < 0.05.

The sample recruited for this study was powered to detect a significant difference in the IKDC between ACLR patients and the MLKR group that injured their knee specifically during pivoting sports at 24 months. A minimal clinically important difference (MCID) of 9.0 has been reported for the IKDC in patients undergoing ACLR [1]. Using G‐power, to determine a between‐group difference of the proposed MCID with an SD of 7 based on pre‐existing data in ACLR patients at 24 months, at least 14 patients in each of these groups were required to have 95% power in detecting a significant difference in the IKDC between groups.

## RESULTS

### Patient demographics, injury and surgery characteristics

Table 1 shows the patient demographics, injury and surgery characteristics, of the ACLR (*n* = 50), full MLKR (*n* = 50) and pivoting sport MLKR (*n* = 20) cohorts. The nature of the multiligamentous injuries is provided for the MLKR cohorts as per the Schenck Classification, whereas the specific surgical ligamentous combinations are shown in Table 1.

### Subjective outcomes and activity

**Figure 1:**
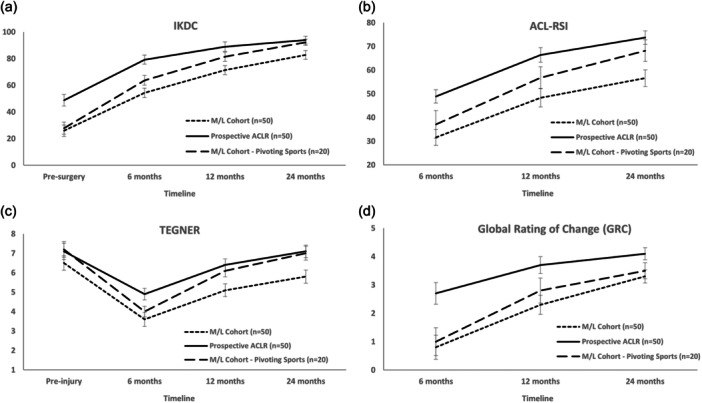
Graphical representation of the full ACLR and MLKR cohorts, as well as the MLKR cohort that injured themselves participating in pivoting sports, demonstrating group changes from presurgery (where relevant) to 6, 12 and 24 months postsurgery, as well as differences between groups, for the (a) International Knee Documentation Committee (IKDC) form, (b) anterior cruciate ligament return to sport after injury score (ACL‐RSI), (c) Tegner activity scale and (d) global rating of change (GRC) scale. ACLR, anterior cruciate ligament reconstruction; MLKR, multiligamentous knee reconstruction.

Within the full MLKR cohort, the group comparisons in PROMs for the Schenck classification (KDI versus KDII‐V) and CPN involvement (with versus without CPN involvement) are shown in Tables 3 and 4, respectively. Group comparisons (ACLR, full MLKR and pivoting sport MLKR cohorts) for PROMs are shown in Table 5. The full MLKR cohort reported significantly lower IKDC scores than the ACLR cohort preoperatively (*p* < 0.001) and at 6 (*p* < 0.001), 12 (*p* < 0.001) and 24 (*p* = 0.008) months. There were no differences between the ACLR cohort and the pivoting sport MLKR cohort at 12 (n.s.) or 24 (n.s.) months (Figure 1a). The full MLKR cohort reported significantly worse ACL‐RSI scores than the ACLR cohort at 6 (*p* < 0.001), 12 (*p* < 0.001) and 24 (*p* < 0.001) months (Figure 1b). At 24 months, there was no difference (n.s) in the ACL‐RSI between the ACLR and pivoting sports MLKR cohorts (Figure 1b). The full MLKR cohort reported a significantly lower TAS than the ACLR cohort at 6 (*p* = 0.005), 12 (*p* = 0.015) and 24 (*p* < 0.001) months, though no 12 (n.s.) and 24 (n.s.) month differences were seen between the ACLR and pivoting sports MLKR cohorts (Figure 1c). There were no GRC differences (n.s.) between the full and pivoting sports MLKR cohorts at any timepoint, though both MLKR groups reported a lower (*p* < 0.05) GRC when compared with the ACLR cohort at all timepoints (Figure 1d). Satisfaction responses for all groups are reported in Table 6.

**Table 3:** PROMs throughout the pre‐ and postoperative timeline for the patients in the full MLKR cohort, based on Schenck classification (KDI versus KDII–V).

Timepoint	Group	IKDC	KOS	Lysholm	Cincinnati	Tegner*	GRC	ACL‐RSI
Presurgery	MLKR (KDI)	27.7 (17.4)	31.7 (17.3)	33.7 (22.3)	34.0 (22.9)	6.1 (1.8)	NA	NA
	MLKR (KDII‐V)	21.3 (10.6)	23.2 (17.5)	30.4 (21.2)	20.2 (14.4)	7.5 (3.6)		
6 months	MLKR (KDI)	52.7 (17.8)	59.1 (10.4)	72.3 (15.0)	64.8 (16.4)	3.6 (1.1)	0.5 (2.8)	32.9 (23.4)
	MLKR (KDII‐V)	55.5.3 (16.7)	60.9 (16.8)	73.9 (24.5)	67.0 (21.9)	3.6 (1.2)	1.2 (3.3)	29.1 (24.4)
12 months	MLKR (KDI)	70.7 (18.6)	67.2 (12.9)	82.4 (17.1)	80.3 (15.9)	5.0 (1.3)	2.4 (2.2)	45.4 (28.8)
	MLKR (KDII‐V)	74.3 (11.7)	70.5 (8.4)	85.6 (10.1)	81.6 (12.6)	5.4 (1.5)	2.1 (2.9)	54.6 (23.6)
24 months	MLKR (KDI)	83.6 (8.8)	71.3 (6.6)	86.8 (10.7)	85.8 (11.2)	5.7 (1.4)	3.4 (1.6)	55.0 (25.1)
	MLKR (KDII‐V)	82.5 (11.1)	74.6 (5.4)	91.5 (7.9)	88.5 (8.2)	6.0 (2.3)	3.1 (1.7)	58.4 (25.3)
Time effect (*p* value)		<0.0001	<0.0001	<0.0001	<0.0001	<0.0001	<0.0001	<0.0001
Group effect (*p* value)		n.s.	n.s.	n.s.	n.s.	n.s.	n.s.	n.s.
Interaction effect (*p* value)		n.s.	n.s.	n.s.	n.s.	n.s.	n.s.	n n.s.

*Note*: Values are shown as means (standard deviation).

Abbreviations: ACL‐RSI, Anterior Cruciate Ligament Return to Sport after Injury Score; GRC, Global Rating of Change; IKDC, International Knee Documentation Committee Subjective Knee Evaluation Form; KD, knee dislocation; KOS, Knee Outcome Survey; NA, not applicable; n.s., not significant; PROMs, patient‐reported outcome measures.

* Tegner is preinjury, as opposed to presurgery.

**Table 4:** PROMs throughout the pre‐ and postoperative timeline for the patients in the full MLKR cohort, based on Schenck classification (KDI versus KDII–V).

Timepoint	Group	IKDC	KOS	Lysholm	Cincinnati	Tegner*	GRC	ACL‐RSI
Presurgery	MLKR (no CPN)	25.0 (13.2)	29.5 (16.0)	33.5 (20.4)	31.0 (20.8)	6.6 (1.9)	NA	NA
	MLKR (CPN)	26.2 (27.9)	28.7 (25.9)	29.0 (29.8)	25.3 (26.7)	6.0 (2.2)		
6 months	MLKR (no CPN)	52.8 (17.1)	58.6 (12.8)	71.7 (18.1)	63.3 (18.0)	3.6 (1.2)	0.5 (3.2)	29.2 (17.9)
	MLKR (CPN)	57.8 (18.5)	64.0 (12.3)	77.3 (20.6)	74.3 (17.8)	3.8 (0.8)	1.8 (1.3)	38.5 (35.0)
12 months	MLKR (no CPN)	71.1 (17.8)	67.7 (12.4)	83.4 (16.3)	80.2 (15.4)	5.1 (1.3)	2.0 (2.6)	46.5 (27.2)
	MLKR (CPN)	72.3 (82.2)	70.2 (9.0)	83.7 (11.7)	82.3 (13.0)	5.3 (1.5)	3.3 (1.0)	50.0 (28.4)
24 months	MLKR (no CPN)	82.8 (8.5)	71.7 (6.6)	87.1 (10.8)	86.8 (10.7)	5.8 (1.6)	3.1 (1.8)	55.3 (24.9)
	MLKR (CPN)	82.6 (11.8)	73.8 (5.5)	89.8 (9.4)	88.2 (10.9)	5.8 (1.9)	3.4 (1.0)	58.4 (26.2)
Time effect (*p* value)		<0.0001	<0.0001	<0.0001	<0.0001	<0.0001	<0.0001	<0.0001
Group effect (*p* value)		n.s.	n.s.	n.s.	n.s.	n.s.	0.007	n.s.
Interaction effect (*p* value)		n.s.	n.s.	n.s.	n.s.	n.s.	n.s.	n.s.

*Note*: Values are shown as means (standard deviation).

Abbreviations: ACL‐RSI, Anterior Cruciate Ligament Return to Sport after Injury Score; CPN, common peroneal nerve; GRC, Global Rating of Change; IKDC, International Knee Documentation Committee Subjective Knee Evaluation Form; KD, Knee dislocation; KOS, Knee Outcome Survey; MLKR, multiligamentous knee reconstruction; NA, not applicable; n.s., not significant; PROMs, patient‐reported outcome measures.

* Tegner is preinjury, as opposed to presurgery.

**Table 5:** PROMs throughout the pre‐ and postoperative timeline for the patients in the full ACLR and MLKR cohorts, as well as the MLKR cohort that injured themselves participating in pivoting sports.

Timepoint	Group	IKDC	KOS	Lysholm	Cincinnati	Tegner*	GRC	ACL‐RSI
Presurgery	MLKR	25.9 (15.8)	29.4 (17.3)	32.8 (21.7)	30.2 (21.5)	6.5 (1.9)	NA	NA
	MLKR (pivoting)	27.9 (22.8)	31.1 (18.4)	31.3 (23.6)	33.9 (28.9)	7.2 (2.0)		
	ACLR	48.8 (16.5)	52.7 (13.1)	55.6 (19.4)	50.5 (17.8)	7.1 (1.5)		
6 months	MLKR	54.3 (17.3)	59.7 (12.7)	72.9 (18.4)	65.6 (18.1)	3.6 (1.1)	0.8 (2.9)	31.6 (23.4)
	MLKR (pivoting)	63.7 (18.3)	64.6 (9.2)	84.1 (11.4)	75.4 (11.2)	4.0 (1.3)	1.0 (2.9)	37.1 (29.1)
	ACLR	79.2 (9.5)	72.8 (5.2)	87.5 (8.2)	82.7 (8.4)	4.9 (1.1)	2.7 (1.2)	48.9 (19.9)
12 months	MLKR	71.4 (16.7)	68.3 (11.6)	83.4 (15.1)	80.7 (14.7)	5.1 (1.3)	2.3 (2.4)	48.3 (27.0)
	MLKR (pivoting)	81.4 (16.8)	73.6 (6.0)	91.3 (6.2)	88.5 (8.9)	6.1 (1.1)	2.8 (2.2)	56.8 (32.8)
	ACLR	88.9 (8.9)	76.3 (3.5)	93.8 (5.9)	91.8 (7.3)	6.4 (1.5)	3.7 (0.7)	66.4 (21.3)
24 months	MLKR	82.8 (9.2)	72.3 (6.3)	87.8 (10.3)	87.2 (10.5)	5.8 (1.7)	3.3 (1.6)	56.6 (24.7)
	MLKR (pivoting)	92.2 (6.7)	74.1 (6.3)	92.1 (9.0)	91.2 (9.8)	7.0 (1.3)	3.5 (2.2)	68.2 (22.9)
	ACLR	93.9 (6.4)	77.7 (4.1)	96.5 (4.6)	96.1 (5.3)	7.1 (1.5)	4.1 (1.0)	73.7 (19.5)
Time effect (*p* value)		<0.0001	<0.0001	<0.0001	<0.0001	<0.0001	<0.0001	<0.0001
Group effect (*p* value)		<0.0001	<0.0001	<0.0001	<0.0001	0.001	0.001	0.008
Interaction effect (*p* value)		n.s.	n.s.	0.035	n.s.	n.s.	n.s.	n.s.

*Note*: Values are shown as means (standard deviation).

Abbreviations: ACLR, anterior cruciate ligament reconstruction; ACL‐RSI, Anterior Cruciate Ligament Return to Sport after Injury Score; GRC, Global Rating of Change; IKDC, International Knee Documentation Committee Subjective Knee Evaluation Form; KD, Knee dislocation; KOS, Knee Outcome Survey; MLKR, multiligamentous knee reconstruction; NA, not applicable; n.s., not significant; PROMs, patient‐reported outcome measures.

* Tegner is preinjury, as opposed to presurgery.

**Table 6:** Satisfaction gradings (very satisfied, somewhat satisfied, somewhat dissatisfied, very dissatisfied) for each of the five satisfaction items, specifically at 24 months postsurgery, for the full ACLR and MLKR cohorts, as well as the MLKR cohort that injured themselves participating in pivoting sports.

Group	Satisfaction item	Pain relief	Improving ability to undertake ADLs	Improving ability to participate in recreational activities	Improving ability to participate in sport	Overall satisfaction
MLKR (*n* = 50)	Very satisfied	19	30	23	13	19
	Satisfied	19	12	17	21	21
	Dissatisfied	7	4	5	9	5
	Very dissatisfied	5	4	5	7	5
	Satisfied overall, *n* (%)	38 (76.0)	42 (84.0)	40 (80.0)	34 (68.0)	40 (80.0)
MLKR (pivoting) (*n* = 20)	Very satisfied	13	16	9	8	12
	Satisfied	5	3	8	8	5
	Dissatisfied	2	1	2	4	3
	Very dissatisfied	0	0	1	0	0
	Satisfied overall, *n* (%)	18 (90.0)	19 (95.0)	17 (85.0)	16 (80.0)	17 (85.0)
ACLR (*n* = 50)	Very satisfied	43	44	43	36	42
	Satisfied	6	6	6	11	7
	Dissatisfied	1	0	1	3	1
	Very dissatisfied	0	0	0	0	0
	Satisfied overall, *n* (%)	49 (98.0)	50 (100.0)	49 (98.0)	47 (94.0)	49 (98.0)

Abbreviations: ACLR, anterior cruciate ligament reconstruction; ADLs, activities of daily living; MLKR, multiligamentous knee reconstruction.

### Physical measures

**Figure 2:**
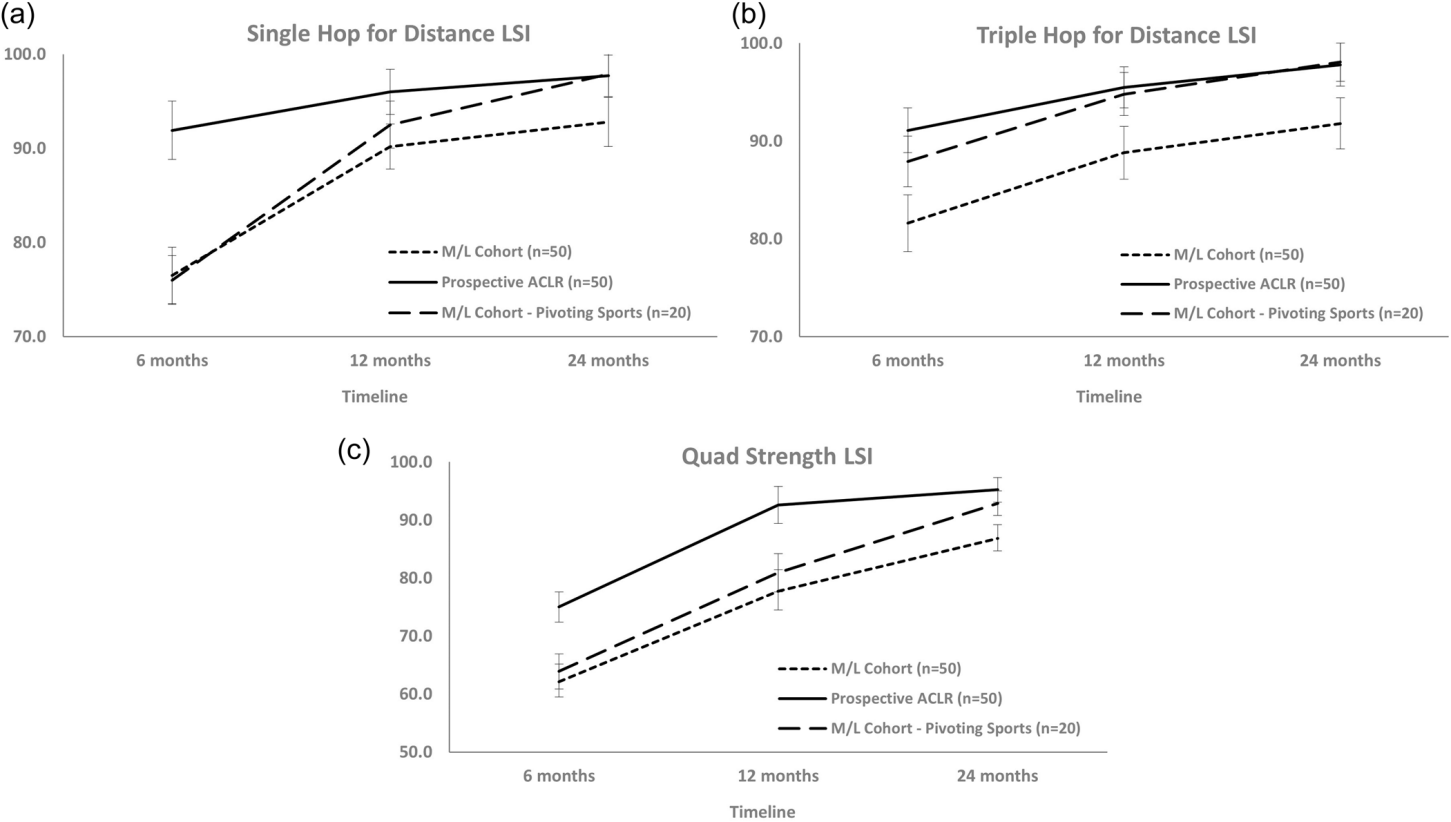
Graphical representation of the full ACLR and MLKR cohorts, as well as the MLKR cohort that injured themselves participating in pivoting sports, demonstrating group changes over the 6‐, 12‐ and 24‐month postoperative period, as well as differences between groups, for the (a) single hop for distance LSI, (b) triple hop for distance LSI and (c) quadriceps strength LSI. ACLR, anterior cruciate ligament reconstruction; LSI, limb symmetry index; MLKR, multiligamentous knee reconstruction.

Objective measures are shown in Table 7. SHD LSIs for the full MLKR cohort were lower than the ACLR group at 6 (*p* < 0.001) and 12 (*p* = 0.002) months (Figure 2a). The ACLR group only demonstrated significantly higher SHD LSIs to the pivoting sport MLKR cohort at 6 months (*p* < 0.001) (Figure 2a). THD LSIs for the ACLR group were higher than the full MLKR group at 6 (*p* < 0.001), 12 (*p* < 0.001) and 24 (*p* < 0.001) months, though no differences (n.s.) were seen between the ACLR and pivoting sports MLKR groups (Figure 2b). Peak knee extensor strength LSIs were significantly lower for the full MLKR cohort versus the ACLR group at 6 (*p* < 0.001), 12 (*p* < 0.001) and 24 (*p* < 0.001) months (Figure 2c). No differences (n.s.) were seen at 24 months between the ACLR and pivoting sports MLKR groups (Figure 2c).

**Table 7:** Active knee range of motion and LSIs for hop tests and peak isokinetic knee extensor (quadriceps) and flexor (hamstring) torque, for the full ACLR and MLKR cohorts, as well as the MLKR cohort that injured themselves participating in pivoting sports at 6, 12 and 24 months postsurgery.

Timepoint	Group	Knee flexion (°)	Knee extension (°)	Knee extensor torque LSI	Knee flexor torque LSI	SHD LSI	THD LSI
6 months	MLKR	128.5 (13.2)	2.0 (3.6)	62.1 (15.2)	92.5 (12.6)	76.5 (13.8)	81.6 (17.6)
	MLKR (pivoting)	134.8 (7.7)	0.3 (4.5)	63.9 (13.3)	95.1 (8.7)	76.0 (13.2)	87.9 (8.2)
	ACLR	138.8 (8.0)	0.5 (1.8)	75.0 (18.1)	95.8 (14.0)	91.9 (7.7)	91.1 (9.2)
12 months	MLKR	136.3 (8.6)	0.3 (3.0)	77.7 (13.2)	97.2 (13.1)	90.2 (9.6)	88.8 (12.1)
	MLKR (pivoting)	139.7 (6.9)	−0.5 (4.1)	80.9 (10.0)	99.2 (13.1)	92.5 (4.2)	94.8 (4.4)
	ACLR	142.2 (7.9)	−0.1 (2.1)	92.6 (10.0)	98.3 (13.6)	96.0 (6.9)	95.5 (7.5)
24 months	MLKR	139.5 (7.2)	−0.3 (2.6)	86.8 (10.4)	97.0 (9.1)	92.8 (11.4)	91.8 (11.1)
	MLKR (pivoting)	143.1 (4.6)	−1.2 (3.7)	92.9 (5.2)	99.5 (5.9)	97.9 (2.5)	98.1 (4.2)
	ACLR	144.5 (7.0)	−1.0 (2.1)	95.2 (10.8)	98.7 (9.3)	97.7 (5.0)	97.8 (5.1)
Time effect (*p* value)		<0.0001	<0.0001	<0.0001	0.041	<0.0001	<0.0001
Group effect (*p* value)		0.003	n.s.	<0.0001	n.s.	<0.0001	0.023
Interaction effect (*p* value)		n.s.	n.s.	n.s.	n.s.	0.040	n.s.

*Note*: Values are shown as means (standard deviation).

Abbreviations: ACLR, anterior cruciate ligament reconstruction; LSIs, limb symmetry indices; MLKR, multiligamentous knee reconstruction; n.s., not significant; SHD, single hop for distance; THD, triple hop for distance.

## DISCUSSION

The most important findings of the current study were that superior outcomes were seen to 24 months in patients undergoing ACLR compared with a heterogenous MLKR cohort. However, in patients who sustained their injury during pivoting sports, 24‐month clinical outcomes, function and level of sport were similar between those undergoing ACLR and MLKR that were largely KDI grades (ACLR or PCLR, combined with concomitant MCL, PLC or PMC reconstruction).

For the IKDC, commonly reported in patients after MLKR [9, 12, 23, 24, 34, 36], whereas the ACLR cohort reported better scores than the full MLKR cohort up until 24 months, they only reported better scores at 6 months when compared with the pivoting sport MLKR group. This suggests that while perceived functional capacity is delayed, MLKR patients injured during pivoting sports progress in a similar way to ACLR patients from 12 months. This delay may be associated with slower recovery in physical capacity, a delayed and/or more conservative early rehabilitation progression, or that they may perceive their injury to be more traumatic. Although Levy et al. [24]. reported higher 2‐year IKDC and Lysholm scores after MLKR in those ≤30 versus >30 years of age, the mean scores reported were less than those observed for the MLKR cohort in the current study. LaPrade et al. [22] reported outcomes between patients undergoing double‐bundle PCLR as an isolated or combined procedure, with those undergoing ACLR. They reported no differences in PROMs between isolated PCLR and PCL‐based MLKR, nor any differences between those undergoing isolated PCLR and ACLR. Finally, outcomes from the Danish Knee Ligament Reconstruction Registry [30] reported that PROMs at 12 months postsurgery improved more in those undergoing isolated ACLR versus a cohort of patients undergoing MLKR, of which 78% were Schenck KDI, similar to the current study.

Psychological readiness to RTS remained inferior in the pivoting sport MLKR (compared to ACLR) at 6 and 12 months. Webster et al. [38] reported that a greater SHD LSI in ACLR patients had a positive effect on psychological readiness. However, the lower (and lingering) ACL‐RSI scores in the current study may still be associated with the significant nature of the patient's injury and the ongoing memory of the incident, along with the persistent lack of knee extensor symmetry at 12 months. The association between psychological recovery and knee function has been documented, with lower levels of self‐efficacy linked with lower recovery of muscle function after ACLR [4, 7, 8, 28, 31]. To the best of our knowledge, the ACL‐RSI has not been reported previously after MLKR, especially for those who are more active and have a desire to return to sports. The first hypothesis was only partially supported. Inferior PROMs were seen in the full MLKR versus ACLR cohort. The recovery in these PROMs in the pivoting sport MLKR was generally similar to ACLR patients by 12 months (apart from the ACL‐RSI).

Although the TAS was lower for the full MLKR cohort at all postoperative time points compared with the ACLR cohort, it was only lower for the pivoting sport MLKR cohort at 6 months. A systematic review and meta‐analysis reported that better postoperative TAS scores were observed in patients undergoing MLKR through a low‐energy (during sports, activities of daily living and low falls) versus high‐energy (motor vehicle accidents and high falls) injury mechanism [10]. The similar reported activity level between the ACLR and pivoting sport MLKR cohorts at 12 and 24 months, was in support of the second hypothesis. Bakshi et al. [5] reported that the return to play rate after MLKI was significantly lesser than those after ACL injuries, in elite‐level National Football League athletes. Despite the similarity in the reported activity profile, PROMs and objective hop and strength LSIs, between the ACLR and pivoting sport MLKR patients by 24 months in the current study, it was interesting to note that the ACLR group still reported a higher GRC at 24 months. This highlights the multifaceted nature of overall perceived recovery, including variables beyond what was collected in the current study.

In evaluating the recovery of hop and knee extensor strength symmetry, the ACLR (versus full MLKR) cohort demonstrated significantly higher LSIs for the SHD at 6 and 12 months, and THD and knee extensor strength at all timepoints. In comparison, the ACLR (versus pivoting sport MLKR) cohort only demonstrated a significantly higher SHD LSI at 6 months, and knee extensor strength LSI at 6 and 12 months. These results partially supported the third hypothesis. Research has highlighted an increased reinjury risk in patients not meeting 90% LSI criteria (particularly in peak quadriceps strength and hop capacity) [17, 21]. While the importance of restoring limb performance symmetry after MLKR has been outlined, inclusive of isokinetic strength and hop capacity [29], published evidence has failed to report on the physical recovery of these measures, with studies generally focused on PROMs. In the limited studies available, persistent strength deficits after MLKR at or beyond 24 months have been reported [11, 14, 20]. Gigliotakaes et al. [14] reported persistent isokinetic knee extensor deficits following a two‐stage bicruciate procedure, while Denti et al. [11] also reported 24‐month isokinetic knee flexor and extensor strength deficits in patients who underwent single‐stage bicruciate reconstruction.

Several limitations are acknowledged in this study. First, a paucity of evidence is available reporting recovery of physical performance symmetry after MLKR, making comparison with existing evidence challenging. Second, while the groups were similar for variables such as age, body mass index and sex, we were unable to match for a range of other potentially contributing variables such as the sport, skill level, preinjury training history and conditioning. Third, rehabilitation and RTS guidance were not standardized. We acknowledge that clinical outcomes can be affected by a range of other variables which were not tightly controlled for, such as the quality and duration of rehabilitation, with rehabilitation progressed on a patient‐by‐patient basis under the direction of the patient's own therapist. We do not have information on rehabilitation quality, as well as the frequency and adherence to rehabilitation, which may have been different between those undergoing ACLR versus MLKR. Admittedly, while a multidisciplinary approach was adopted this would more likely be the case given the differing nature of the two procedures and more significant nature of the MLKR (versus ACLR), with the study comparing two different surgical cohorts with rehabilitation pathways that support each surgery.

Fourth, we chose to include concomitant meniscal repair, which was observed in 48% of MLKRs (and 50% of MLKRs injured during pivoting sports) and 46% of ACLRs. While we acknowledge this may be seen as a confounding variable, given they are undertaken so frequently in those undergoing either procedure, as well as the fact that excluding those that underwent meniscal repair in the MLKR would have significantly reduced the cohort size which is presenting less frequently, we included them in both groups. Finally, we acknowledge the heterogeneous nature of the MLKR cohort, a function of patient presentation and the author's goal to include a cohort that was representative of a true MLKR cohort. We appreciate that the majority of the MLKR included were KDI, and we also chose to include the cases that had concomitant CPN involvement, as well as other concomitant issues such as osteochondral autograft transplantation and tibial plateau fracture, again a reflection of this patient cohort presenting in clinical practice. It should also be noted that in comparing PROMs between KDI and KDII‐V cohorts, as well as those with or without CPN involvement, no statistical group differences were identified.

As the clinical relevance, patients that undergo MLKR following injury during pivoting sports, albeit in a biased KDI cohort that underwent ACLR or PCLR combined with concomitant MCL, PLC or PMC reconstruction, demonstrate similar clinical, functional and RTS outcomes when compared with those undergoing ACLR. The outcomes of this study are important to orthopaedic teams in counselling patients on recovery and RTS expectations after MLKR.

## CONCLUSIONS

The current study demonstrated that PROMs, physical recovery and the activity profile were superior up until, and including, 24 months postsurgery in patients undergoing ACLR when compared with a heterogenous MLKR cohort. However, in patients that sustained their injury specifically during pivoting sports, 24‐month clinical outcomes, function and level of sport are similar between those undergoing ACLR and an MLKR cohort that was largely KDI grades (i.e. ACLR or PCLR, combined with concomitant MCL, PLC or PMC reconstruction). The outcomes presented are important to orthopaedic teams in counselling patients on the capacity and associated timeframe of recovery after MLKR, whereas allowing for more optimistic expectations than might previously have been provided after this type of injury.

## AUTHOR CONTRIBUTIONS

*Conceived and designed the study*: Jay R. Ebert, Peter K. Edwards, Satyen Gohil and Peter D'Alessandro. *Supervised the conduct of the study*: Jay R. Ebert, Peter K. Edwards, Alistair I. W. Mayne, Peter S. E. Davies, Robert Evans, Randeep S. Aujla, Shahbaz S. Malik, Stephen Dalgleish, Satyen Gohil and Peter D'Alessandro. *Analysed the data*: Jay R. Ebert, Peter K. Edwards, Alistair I. W. Mayne and Peter S. E. Davies. *Wrote the initial drafts*: Jay R. Ebert, Peter K. Edwards and Peter S. E. Davies. *Critically revised the manuscript*: Jay R. Ebert, Peter K. Edwards, Alistair I. W. Mayne, Peter S. E. Davies, Robert Evan, Randeep S. Aujla, Shahbaz S. Malik, Stephen Dalgleish, Satyen Gohil and Peter D'Alessandro. *Ensure the accuracy of the data and analysis*: Jay R. Ebert, Peter K. Edwards, Alistair I. W. Mayne, Peter S. E. Davies, Robert Evans, Randeep S. Aujla, Shahbaz S. Malik, Stephen Dalgleish, Satyen Gohil and Peter D'Alessandro. I confirm that all authors have seen and agree with the contents of the manuscript and agree that work has not been submitted or published elsewhere in whole or part.

## CONFLICT OF INTEREST STATEMENT

The authors declare no conflict of interest.

## ETHICS STATEMENT

Ethics approval was obtained by the University of Western Australia (RA/4/20/4112). Informed and written consent was obtained from all individual participants included in the study.

## Data Availability

Data have not been made publicly available, though data sets generated during the current study can be made available from the corresponding author on reasonable request.
